# Hashimoto's Thyroiditis and Graves' Disease in One Patient: The Extremes of Thyroid Dysfunction Associated with Interferon Treatment

**DOI:** 10.1155/2016/6029415

**Published:** 2016-03-02

**Authors:** R. H. Bishay, R. C. Y. Chen

**Affiliations:** ^1^Department of Endocrinology and Metabolism, Concord Repatriation General Hospital, Concord, NSW 2139, Australia; ^2^Concord Clinical School, Sydney Medical School, University of Sydney, Sydney, NSW 2005, Australia

## Abstract

Autoimmune thyroid disease associated with interferon therapy can manifest as destructive thyroiditis, Graves' Hyperthyroidism, and autoimmune (often subclinical) hypothyroidism, the latter persisting in many patients. There are scare reports of a single patient developing extremes of autoimmune thyroid disease activated by the immunomodulatory effects of interferon. A 60-year-old man received 48 weeks of pegylated interferon and ribavirin therapy for chronic HCV. Six months into treatment, he reported fatigue, weight gain, and slowed cognition. Serum thyroid stimulating hormone (TSH) was 58.8 mIU/L [0.27–4.2], fT4 11.1 pmol/L [12–25], and fT3 4.2 pmol/L [2.5–6.0] with elevated anti-TPO (983 IU/mL [<35]) and anti-TG (733 U/mL [<80]) antibodies. He commenced thyroxine with initial clinical and biochemical resolution but developed symptoms of hyperthyroidism with weight loss and tremor 14 months later. Serum TSH was <0.02 mIU/L, fT4 54.3 pmol/L, and fT3 20.2 pmol/L, with an elevated TSH receptor (TRAb, 4.0 U/L [<1.0]), anti-TPO (1,163 IU/mL) and anti-TG (114 U/mL) antibodies. Technetium scan confirmed Graves' Disease with bilateral diffuse increased tracer uptake (5.9% [0.5–3.5%]). The patient commenced carbimazole therapy for 6 months. Treatment was ceased following spontaneous clinical and biochemical remission (TSH 3.84 mIU/L, fT4 17pmol/L, fT3 4.5 pmol/L, and TRAb <1 U/L). This raises the need to monitor thyroid function closely in patients both during and following completion of interferon treatment.

## 1. Background

Approximately 3% of the world population, or 180 million people, are infected with hepatitis C virus (HCV) and 38–76% will have at least one extrahepatic manifestation [1]. In a large cohort of US adults with HCV, a small but appreciable proportion will develop clinically significant autoimmune thyroid disease (AITD) (adjusted HR 1.13), making AITD the commonest endocrinopathy in HCV patients [2].

Exogenous exposure to interferon- (IFN-) based therapies has long been known to have a predilection for causing AITD. The IFNs are a family of cytokine proteins produced by white blood cells, fibroblasts, and cells of the adaptive immune system. Congruent with their name, they interfere with viral replication among other functions. There are three main groups of IFN, namely, alpha (*α*), beta (*β*), and gamma (*γ*). Interferon-*α* is commonly used for its clinical ability to alter the immune response in a variety of conditions such as HCV and multiple sclerosis. Prior to the advent of directly acting antiviral drugs (DAAs), combination of pegylated IFN-*α* and ribavirin therapy remained the gold standard for treatment of patients with chronic HCV infection; however in many parts of the world (including Australia) IFN-*α* based therapies are still being used [3]. Numerous reports of AITD have been reported in HCV patients in the setting of current or following IFN-based treatment [4–6]. Data from three studies on 421 patients who were antibody negative prior to IFN-*α* therapy showed anti-TPO positivity in 9.5%, and over half (58%) of those patients developed overt AITD. Overall, pooling the incident rates from six studies, AITD seems to affect 2.7 to 10%, or an average of 6%, of IFN-*α* treated patients [6].

Hypothyroidism is the dominant form of thyroid dysfunction but studies vary on its incidence, from 66 to 97% of cases [6]. Furthermore, over 87% of hypothyroid patients are also positive for anti-TPO antibodies, reflecting its basis as an autoimmune process. Importantly, autoimmune hypothyroidism may persist in 56 to 59% of patients. Incidence of hyperthyroidism varies among studies as well, with approximately 25% to 60% suffering from transient thyrotoxicosis and the remainder having scintigraphic and/or biochemical evidence of Graves' Hyperthyroidism, many of which required treatment [6]. In contrast, a large study of 869 HCV patients receiving IFN-*α* reported biphasic thyroiditis responsible for the majority of AITD cases (58%) [7].

With some exceptions, there are very few case reports reporting extremes of AITD in a single patient in association with IFN-*α* treatment [8, 9]. The “swinging thyroid” concept was illustrated in two recent cases, where the characteristic biphasic pattern of thyroiditis, initial TRAb-negative thyrotoxicosis with subsequent development of clinical and biochemical hypothyroidism, was then followed by biochemical and scintigraphic evidence of Graves' Hyperthyroidism [8]. To date, there are scarce reports documenting the development of initial clinical and biochemical hypothyroidism associated with high titres of anti-TPO with subsequent development of Graves' Disease in a single patient, illustrating a novel clinical pattern of AITD. This case also highlights the importance of understanding the pathophysiological mechanism underpinning the unpredictable course of autoimmune disease associated with IFN-*α*, as discussed below.

## 2. Case Presentation

A 60-year-old man originally from China with no prior history of thyroid or autoimmune disease received a standard course of 48-week pegylated IFN-*α* and ribavirin therapy for chronic HCV (genotype 1b) with achievement of sustained virological response. He had compensated chronic liver disease with cirrhosis (Child-Pugh A) without other complications and no other reported medical problems. He denied previous use of amiodarone, lithium, medications, or supplements containing iodine or exposure to contrast. He was a nonsmoker and did not drink alcohol. Six months after commencing treatment, he reported fatigue, weight gain of 3 kg, and slowed cognition at a routine clinical visit. He denied changes to his skin, hair, or bowel habits. On examination, his vital signs were normal. His weight was 78 kg with a body mass index (BMI) of 26.8 kg/m^2^. There was no goitre and there were no signs of oedema, dermopathy, ophthalmopathy, or lymphadenopathy. Cardiac, respiratory, and gastrointestinal examinations were normal and in particular no stigmata of chronic liver disease was present. A peripheral neurological examination including reflexes was normal.

**Figure 1:**
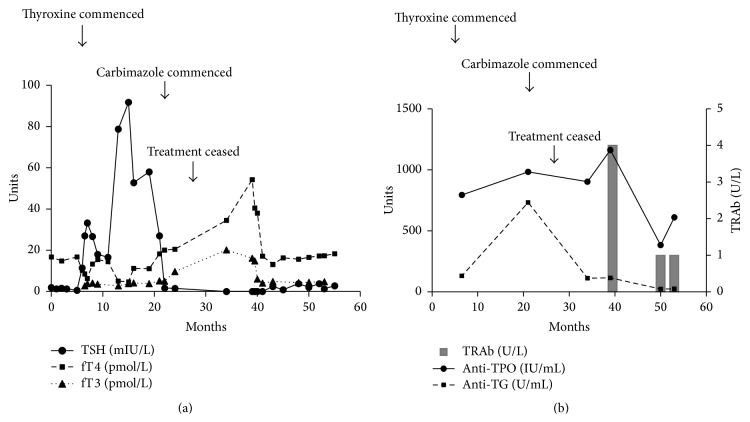
(a) Temporal pattern of thyroid function tests showing both hypothyroidism and subsequent T3-toxicosis, consistent with Graves' Disease. (b) Thyroid antibodies showing persistently elevated anti-TPO and later development of thyrotoxicosis with positive TRAb titres. TSH,* thyroid stimulating hormone*; fT4,* free thyroxine*; fT3,* free triiodothyronine*; anti-TPO,* anti-thyroperoxidase antibody*; anti-TG,* anti-thyroglobulin antibody*; TRAb,* TSH receptor antibody*.

Serum biochemistry including electrolytes, renal function, and full blood count were within normal limits, with no biochemical or serological evidence of decompensated liver disease. Alpha-fetoprotein (AFP) was normal at 3.5 kIU/L [normal, 0.0–6.0]. Pretreatment HCV quantitative RNA was >3,000,000 IU/mL and was undetectable 6 months after commencing treatment. A recent abdominal ultrasound revealed a radiologically normal liver with no signs of portal hypertension or hepatic lesions. His serum thyroid stimulating hormone (TSH) prior to therapy was 1.65 mIU/L [normal, 0.27–4.2] with a normal free T4 (fT4) of 14.5 pmol/L [normal, 12–25]. The TSH at the time of review (i.e., 6 months later) was 58.8 mIU/L, free T4 was 11.1 pmol/L, and free T3 was 4.2 pmol/L [normal, 2.5–6.0] (Figure 1(a)). Anti-TPO (983 IU/mL, normal <35) and anti-TG (733 U/mL, normal <80) antibodies were elevated (Figure 1(b)).

**Figure 2:**
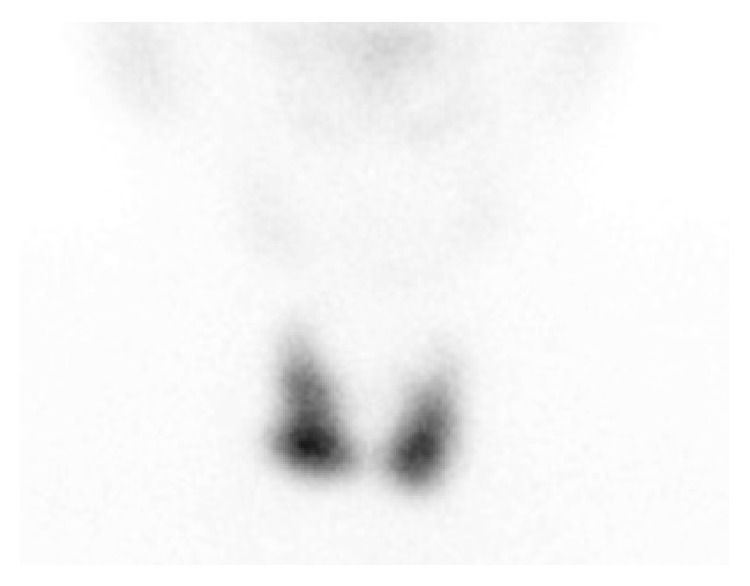
Technetium scan revealing bilateral diffuse increased tracer uptake at 5.9% [normal 0.5–3.5%], consistent with Graves' Disease.

Given his symptoms and biochemical evidence of autoimmune hypothyroidism, the patient was commenced on standard adult thyroxine replacement therapy at 100 mcg daily. His symptoms improved and there was full clinical and biochemical resolution of his hypothyroid state. Fourteen months later, he returned for follow-up reporting symptoms of hyperthyroidism with weight loss, tremor, and palpitations. He had lost 5 kg of weight. At the time of review, serum TSH was suppressed at <0.02 mIU/L with elevated fT4 of 54.3 pmol/L, fT3 of 20.2 pmol/L, and an elevated TRAb of 4.0 U/L (normal <1.0), with anti-TPO (1,163 IU/mL) and anti-TG (114 U/mL) antibodies. Technetium scan revealed bilateral diffuse increased tracer uptake (5.9%, normal 0.5–3.5%), consistent with Graves' Disease (Figure 2).

The patient's thyroxine was ceased and he was placed on antithyroid treatment with carbimazole 5 mg TDS. He self-ceased therapy 6 months later, with serendipitous clinical and biochemical remission. His serum TSH was 3.84 mIU/L, fT4 17 pmol/L, and fT3 4.5 pmol/L, but with persistently elevated anti-TPO at 383 IU/mL. Anti-TG 23 U/mL and TRAb < 1 U/L were normal. At the time of writing, the patient remained clinically and biochemically euthyroid.

## 3. Discussion

Development of hypothyroidism with subsequent Graves' Disease represents a rare and novel clinical pattern of AITD following IFN-*α* therapy. Although IFN-*α* associated AITD has been described for more than 30 years, the immunological mechanisms have only been recently elucidated [8, 10]. This is clinically relevant in many parts of the world, including Australia, where IFN-*α* based therapies are still being used to treat chronic HCV infection. For example, although DAAs are becoming standard of care globally, treatment for HCV genotype 1 (54% of diagnosed cases in the Australian population) involves weekly pegylated IFN injections, with twice-a-day ribavirin tablets and a once-a-day tablet of simeprevir (Olysio). For genotype 3 (37% of the population), the mainstay of therapy is a combination of weekly pegylated IFN injections and daily ribavirin tablets over a period of 26 weeks [3]. Thus, IFN-*α* is still being used countrywide and in many parts of the world, making the adverse effects of IFN-*α* therapy relevant to hepatologists, endocrinologists, and primary care physicians.

Patients with chronic HCV without prior treatment with IFN-*α* already have modulation of the immune system. In particular, the cytotoxic response of CD4 T-cells is primed by high levels of circulating interferon-*γ* (IFN-*γ*) and interleukin-2 (IL-2). When exogenous IFN-*α* is administered, the cytotoxic activation of CD4 T-cells is amplified further, primarily via a Th1 mediated pathway, and interacts with abnormally expressed major histocompatibility complex class I antigen surface expression on thyrocytes. The end result is apoptosis and destruction of thyrocytes and thyroid follicles [8]. Exogenous IFN-*α* may also influence switching to a Th2 pathway, which can result in the development of autoantibodies (e.g., anti-TPO, anti-TG) resulting in thyrotoxicosis, due to thyroid follicular rupture and release of stored thyroid hormones into the circulation. Thyroiditis is confirmed by low pertechnetate uptake scan and positive thyroid autoantibody titres. Thionamides are contraindicated in this scenario given the likelihood of exacerbating thyroid dysfunction, and corticosteroids are ineffective. Following depletion of thyroid hormone reserve, hypothyroidism may ensue and subsequently recover, though many patients will remain hypothyroid [6]. Interestingly, our index patient developed autoimmune hyperthyroidism consistent with Graves' Disease, with positive TRAb-antibody titres, T3-toxicosis, and confirmatory scintigraphy, following initial hypothyroidism. The fact that Graves' Disease, which is thought to be due to a Th2 mediated production of stimulating TRAb, is much less common in IFN-*α* treated patients suggests that IFN-*α* preferentially activates Th1 immunity which in turn leads to the higher rate of destructive thyroiditis seen in these patients. This is consistent with previous reports that IFN-*α* may have suppressive effects on Th2 immunity [11] and supports the results from several studies which illustrate that the predominant form of AITD in IFN-*α* treated patients is indeed autoimmune hypothyroidism [6].

As to why Graves' Disease develops later, even after cessation of IFN-*α*, is unknown. It has been proposed that there is further modulation of the immune system in a genetically susceptible individual via a Th2 mechanism, resulting in the production of TSH stimulating immunoglobulin (TSI) [8]. In contrast to hypothyroidism or biphasic thyroiditis, the Graves' Disease may develop much later after treatment, as in the index patient, who became thyrotoxic 9 months after completing IFN-*α* treatment. Overall, IFN-*α* induced AITD can occur from as early as 4 weeks to as late as 23 months, with a median onset of 17 weeks after commencing IFN-*α* [12]. However, one study failed to reveal a difference with respect to the type of AITD that developed and onset of disease. Furthermore, a small study of 94 patients failed to find a relationship between AITD in IFN-*α* treated patients and pretreatment HCV virological parameters, HCV genotype, total dose of pegylated IFN-*α* or ribavirin, use of nonpegylated IFN-*α*, or virological outcome [10].

Risk factors associated with AITD in the setting of IFN-*α* treatment for HCV include female gender (RR 4.4) as well as the presence of anti-TPO antibodies prior to therapy (RR 3.9); however treatment with IFN-*α* itself has been associated with the* de novo* development of anti-TPO antibodies [6]. Being female and having a higher pretreatment TSH were strongly associated with biphasic thyroiditis, whereas Asian ethnicity and being a current smoker decreased the risk [7]. Arguably, pretreatment thyroid function may be used to predict those who develop AITD but is undoubtedly indicated in all patients about to commence IFN-*α* therapy. Although no clear guidelines are in place, it has been suggested that thyroid function tests be assessed monthly and 6 months following completion of IFN-*α* therapy [8]. Given this case report, it may be suggested that thyroid function tests be assessed for a longer period of time, for up to 2 years, following IFN-*α* therapy. Ultimately, posttherapy surveillance should be individualised based on patient symptoms, personal or family history of thyroid disease, and presence of anti-thyroid antibodies.
